# Comparative Analysis of Standing Postural Control and Perturbation-Induced Muscle Activity in Transtibial and Transfemoral Amputees

**DOI:** 10.3390/jcm14248737

**Published:** 2025-12-10

**Authors:** Mustafa Cem Türkmen, Hüseyin Çelik, Ali İmran Yalçın, Semra Topuz

**Affiliations:** 1 Department of Therapy and Rehabilitation, Disabled Care and Rehabilitation Program, Sabuncuoğlu Serafeddin Health Services Vocational School, Amasya University, 05100 Amasya, Turkey; 2 Department of Therapy and Rehabilitation, Physiotherapy Program, Sungurlu Vocational School, Hitit University, 19030 Corum, Turkey; fzthuseyincelik@gmail.com; 3 Movement Analysis Laboratory, Faculty of Physical Therapy and Rehabilitation, Hacettepe University, 06800 Ankara, Turkey; fzt.aliyalcin@gmail.com (A.İ.Y.); fztsemra@yahoo.com (S.T.)

**Keywords:** transtibial amputation, transfemoral amputation, EMG, postural control, postural reactions, balance

## Abstract

**Background/Objective:** Postural control differs between individuals with lower limb amputation and the general population. Although previous studies examined the effects of unexpected surface perturbations on postural control in individuals with transtibial amputation (TTA) and individuals with transfemoral amputation (TFA), their impact on lower limb muscle activation remains unclear. This study aimed to assess postural control on a stable surface and to evaluate the effects of unexpected surface perturbations on lower limb muscle activation in unilateral TTAs, TFAs, and in a healthy control group (CG). **Methods:** The study included 10 TTAs, 9 TFAs, and 10 healthy controls. Postural control was assessed using a force platform, and lower limb muscle activity was recorded with surface electromyography during unexpected surface perturbations. **Results:** The TFAs showed the highest anteroposterior and lateral postural sway under compliant surface eyes closed and the highest lateral sway under normal surface eyes closed, whereas the CG showed the lowest values (*p* < 0.05). During forward perturbations, rectus femoris (RF) and tibialis anterior (TA) activations were significantly higher than biceps femoris (BF) and medial head of the gastrocnemius (GM) activations, respectively, across all groups (*p* < 0.05). During backward perturbations, GM activations exceeded TA activations in all groups, while BF activations were higher than RF only in TTAs (*p* < 0.05). Significant group effects were found for RF and BF during forward perturbations, and side effects for BF (forward) and RF (backward) activations (*p* < 0.05). **Conclusions:** Postural control responses vary with the level of lower limb amputation. TFAs relied more on visual input during quiet standing, whereas TTAs demonstrated greater reliance on thigh muscle activation during surface perturbations. These findings highlight the need to consider amputation level in balance and rehabilitation programs.

## 1. Introduction

Maintaining an upright standing posture is a fundamental motor action that enables locomotion and many other movement tasks [1]. Standing postural stability is achieved by positioning the center of mass (CoM) over the base of support (BoS). Although the human body is inherently unstable due to both environmental factors and its own movements, postural control strategies, including predictive (anticipatory), reactive (compensatory), and combined forms, are utilized in daily life to maintain the CoM position over the BoS [2,3]. In predictive postural control, the nervous system prepares postural muscles in advance to maintain balance against voluntary movements that may disturb stability, such as reaching, squatting, arm elevation, or stepping [3,4,5,6]. Reactive postural control, on the other hand, functions to regulate unexpected movements of the CoM within or beyond the BoS [4]. Unexpected external perturbations, such as slipping or tripping during walking, being pushed, or a sudden displacement of the supporting surface while standing, elicit reactive postural control responses [4,7].

Postural control strategies in individuals with lower limb amputation differ from those observed in the general population [8]. Several studies have investigated the effects of unexpected surface perturbations induced by anteroposterior or multidirectional platform movements on postural control in amputees [9,10,11,12]. Nevertheless, research examining the influence of such surface perturbations on muscle activation remains limited. Rusaw et al. investigated response latencies in lower limb muscles of individuals with transtibial amputation following perturbations caused by a platform that suddenly rotated upward or downward [13]. To date, no study has examined the effects of unexpected surface perturbations on lower limb muscle activations in individuals with transtibial amputation (TTA) and transfemoral amputation (TFA). Therefore, the present study aimed to assess postural control on a stable surface and to evaluate the effects of unexpected surface perturbations on lower limb muscle activation in unilateral TTAs and TFAs, as well as in a healthy control group (CG). It is expected that the results obtained from this study will provide important information to guide the development of prosthetic technologies, prosthetic design and alignment processes, and rehabilitation programs for individuals with lower limb amputation.

## 2. Materials and Methods

This study was conducted at the Faculty of Physical Therapy and Rehabilitation, Hacettepe University. This study complied with the tenets of the Declaration of Helsinki and was approved by the Institutional Review Board of Hacettepe University (Protocol code: GO 21/1105) and was registered at ClinicalTrials.gov (NCT05301270) on March 18, 2022. Informed consent was obtained from each participant.

### 2.1. Participants

**Figure 1:**
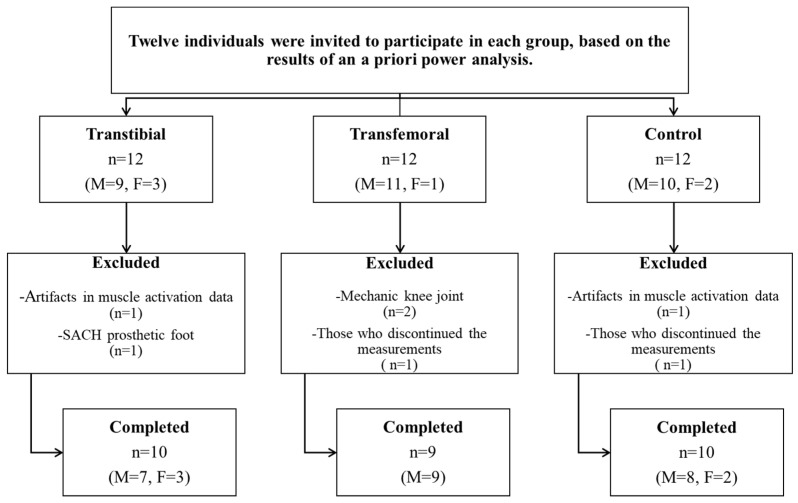
Flowchart of the study. (n: Number of participants, M: Male, F: Female).

An a priori power analysis was conducted using G*Power (version 3.1.9.7, University of Düsseldorf, Germany) based on pilot data from five unilateral TTAs and five unilateral TFAs. Using the means and standard deviations of the anteroposterior postural sway range compliant surface eyes open (Cohen’s d = 1.990) and the forward perturbation intact side (IS) rectus femoris (RF) data (Cohen’s d = 1.162), it was determined that sample sizes of 5 and 10 subjects per group, respectively, would be required to achieve a power of 0.80 at a significance level of 0.05. For this reason, twelve individuals were invited to participate in each of the TTA and TFA groups and an equal number of healthy individuals were invited to form the CG. However, individuals with artifacts in muscle activation data, those using mechanical knee joints or SACH prosthetic feet, and those who discontinued the measurements were excluded from the study. The study was completed with 29 participants: 10 with unilateral TTAs (7 males, 3 females), 9 with unilateral TFAs (9 males), and 10 in the CG (8 males, 2 females) (Figure 1).

The TTA and TFA groups included individuals aged 18–45 who had undergone amputation surgery due to traumatic causes, had been using prostheses for at least one year, had no orthopedic (other than amputation) or neurological problems, and had an activity level of K3 or K4 according to the Medicare Functional Classification Level [14]. The CG consisted of healthy individuals with similar demographic characteristics to those in the TTA and TFA groups and with no orthopedic or neurological problems. Individuals with a body mass index greater than 30 kg/m^2^, musculoskeletal pain, or vestibular, cognitive, or severe visual impairments were excluded from all groups.

### 2.2. Postural Control Assessment

Postural control was assessed using the Bertec Balance Check Screener™ (BP5046, Bertec Co., Columbus, OH, USA), a force platform that measures instantaneous changes in vertical force and center of pressure (CoP). This platform has a firm surface also features a compressible foam surface of the same dimensions, thereby enabling postural control assessments on a soft surface. Participants were asked to place their feet equidistant from the midline of the platform or foam pad and as symmetrically as possible, in a comfortable position of their choice [15]. All participants were evaluated while wearing their usual footwear, since the alignment of the prosthetic foot is adjusted based on the shoes used in daily activities.

The Bertec Balance Check Screener protocols include the Standing Stability tests and the Limits of Stability (LoS) assessment. The Standing Stability tests comprise four static balance assessments: eyes open on a firm surface (normal surface eyes open (NSEO)), eyes closed on a firm surface (normal surface eyes closed (NSEC)), eyes open on a foam surface (compliant surface eyes open (CSEO)), and eyes closed on a foam surface (compliant surface eyes closed (CSEC)). During the Standing Stability tests, participants were instructed to remain as still as possible for 10 s with their arms at their sides. Throughout this period, the postural sway ranges detected by the platform were calculated by the software (Bertec Workbook, version 1.2.0) and recorded in centimeters. These sway ranges serve as indicators of the magnitude of movement in the sagittal and frontal planes. In the LoS assessment, which provides predictions about dynamic balance, participants were instructed to move their body as far as possible forward, backward, right, and left directions, without bending at the trunk and without lifting their feet from the platform. The LoS distances (in centimeters) were calculated by the software (Bertec Workbook, version 1.2.0), representing the maximum CoP path excursion in the four directions. Higher LoS distance values and lower postural sway range values indicate better postural control [15]. All assessments were repeated three times, and the mean of the three repetitions was calculated for each parameter.

For the CoP displacements in the frontal plane derived from the LoS data, measurements were recorded as IS and amputated side (AS) for the TTA and TFA groups, and as dominant side (DS) and nondominant side (NDS) for the CG. In comparisons involving all three groups, IS values were matched with DS, and AS values with NDS. This matching was based on the functional similarity between the dominant role of the IS in maintaining balance and the DS of healthy individuals, as well as the more passive, supportive role of the amputated side and the NDS. Similar matching strategies have been employed in previous studies investigating lateral asymmetry and balance mechanisms in the literature [16,17,18].

### 2.3. Surface Perturbation and Muscle Activation Assessments

The activation of the RF, biceps femoris (BF), tibialis anterior (TA), and medial head of gastrocnemius (GM) muscles in the lower extremities was evaluated using the Delsys Trigno IM wireless surface electromyography (sEMG) system (Delsys Inc., Natick, MA, USA). Data were collected using an eight-channel wireless system equipped with 99.9% Ag surface bar electrodes with a fixed 1 cm inter-electrode distance and a portable data acquisition system. For muscle activation, evaluations were performed with 8 channels for the CG, including RF, BF, TA, and GM in both lower extremities; 6 channels for the TTA group, including RF, BF, TA, and GM in IS and RF and BF muscles in AS; and 4 channels for the TFA group, including RF, BF, TA, and GM in the unaffected side. sEMG signals were recorded with a bandwidth of 20–450 Hz and a common mode rejection ratio (CMRR) of <80 dB at a sampling rate of 1926 Hz.

During the preparation phase, the skin at the electrode placement sites was shaved and cleaned with 70% isopropyl alcohol to minimize skin impedance, and the electrodes were then attached to the skin using double-sided adhesive tape. While cross-talk is a recognized limitation of sEMG systems, electrode placement in the present study was carried out following the recommendations of the SENIAM (Surface Electromyography for the Non-Invasive Assessment of Muscles) guidelines [19,20]. This approach aimed to minimize cross-talk and to ensure standardized electrode placement across all participants. sEMG data were recorded during unexpected surface perturbations while participants stood stationary on a custom-made treadmill capable of moving forward or backward. The custom-made treadmill was set to move over a distance of 14 cm within 0.60 s at an average velocity of 23.33 cm/s [21].

**Figure 2:**
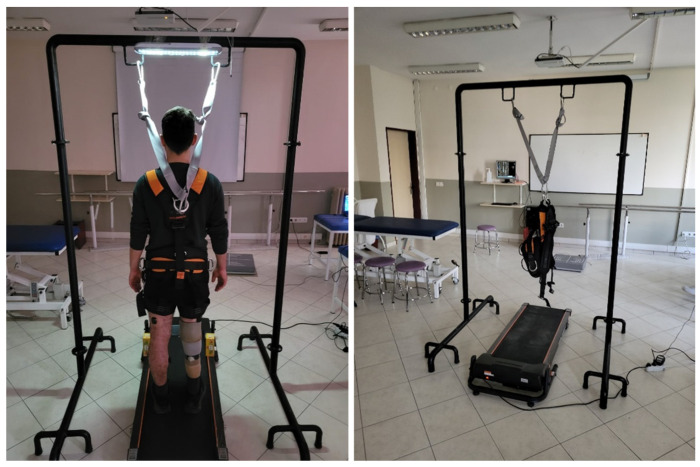
Assessment of muscle activation during surface perturbations.

The measurements were conducted, as in postural control assessments, with participants wearing the footwear they use in daily life. Participants were instructed to place their feet in a self-selected comfortable position symmetrically aligned with the center line of the treadmill and to stand facing forward with their arms relaxed at their sides. To prevent falling and hitting the ground or the treadmill, participants were attached to a steel-framed suspension safety system with a parachute-type harness designed not to support body weight (Figure 2). To minimize excessive fear and/or startle responses that could occur during surface perturbations, a few familiarization trials were conducted before the test. The perturbations in the test protocol were administered in a mixed order (five forward and five backward) based on a random number table ranging from 1 to 10 [22]. This randomization was designed to prevent predictive biomechanical adjustments.

Prior to the initiation of the test protocol, participants were informed that they would not be notified about the timing of the perturbations and that the perturbations would be applied in a random and mixed order. If a participant took a step or experienced a substantial loss of balance, the corresponding perturbation trial was repeated because, under such circumstances, the suspension safety system would support body weight, potentially influencing muscle activation. However, during most parts of the test protocol, participants primarily relied on ankle and hip strategies rather than adopting stepping strategies. Following the completion of the perturbation trials, muscle activity for 6 s during the maximum voluntary isometric contraction (MVIC) of each muscle was measured three times in the specific muscle testing positions that elicited the greatest muscle activation [23]. One-minute rest intervals were provided between trials to minimize fatigue.

### 2.4. Data Analysis

From the raw EMG signals obtained during the surface perturbations, the first and last forward and backward perturbation trials were excluded, and the remaining three forward and three backward perturbation trials were included in the analysis. The raw signals were analyzed using the Delsys EMGworks Analysis software (version 4.7.3.0). Subsequently, to optimize the signal quality and to remove power line noise, a 50 Hz notch filter and filter parameters were set to 4th order Butterworth, band pass filter, cut off frequencies 20–450 Hz, Root Mean Square (RMS) window length 100 ms, window overlap 50 ms. The filtered EMG signals were normalized using MVIC signals and recorded as %MVIC. In normalization, the EMG signals with the highest RMS maximum value out of the three trials were used. The peak values from each trial were recorded, and the mean peak values of the three forward perturbations and the three backward perturbations were calculated.

### 2.5. Statistical Analysis

Normality of the variables was assessed using both visual (histograms and probability plots) and analytical (Shapiro–Wilk tests) methods. Descriptive statistics were presented as frequencies and percentages for categorical variables, and as means with standard deviations for normally distributed numerical variables or as medians with interquartile ranges for non-normally distributed ones. The Monte Carlo exact test and one-way ANOVA were used to compare general physical characteristics among the three groups. The Mann–Whitney U test, Monte Carlo exact test, and Fisher’s exact test were used to compare amputation-related data between the TTA and TFA groups. A one-way ANOVA was conducted to analyze postural control measurements, and the homogeneity of variances was assessed using Levene’s test. When significant differences were found among groups, pairwise post hoc comparisons were performed using Tukey’s test. The Kruskal–Wallis test was used to compare IS/DS muscle activation measurements among groups.

Two mixed-design ANOVAs were performed, followed by Bonferroni-adjusted pairwise comparisons: one to assess side differences in LoS distances between the IS/DS and AS/NDS across the three groups, and the other to examine within-group differences between muscles of the same segment (thigh: RF vs. BF; calf: GM vs. TA) within each group. In addition, a two-way mixed-design ANOVA was conducted with side as the within-subjects factor and group (TTAs and CG) as the between-subjects factor to further assess differences in RF and BF muscle activation. Bonferroni corrections were applied for pairwise comparisons. An α level of *p* < 0.05 was considered statistically significant. All statistical analyses were performed using SPSS software (version 23.0; IBM Corporation, Armonk, NY, USA).

For the post hoc power analysis, comparisons of the anteroposterior postural sway range CSEC, lateral postural sway range NSEC, and lateral postural sway range CSEC among the three groups were used. With statistical significance set at 5% and a total sample size of 30, the post hoc powers (1-β) for these comparisons were found to be 66.46%, 90.02%, and 65.48%, respectively.

## 3. Results

### 3.1. General Physical Characteristics of Participants

The general physical characteristics of all participants are presented in Table 1. It was determined that the groups were similar in terms of sex, age, body height, body mass, body mass index, and intact/dominant limb length, with no statistically significant differences observed among them (*p* > 0.05). However, a significant difference was found in residual/nondominant limb length (*p* < 0.05).

### 3.2. Amputation-Related Data

All participants with amputations were fitted with total surface bearing sockets. Amputation-related data is presented in Table 2. The TTA and TFA groups were statistically similar in terms of amputation age (years), duration of prosthesis use (years), duration of daily prosthesis use (hours), amputation side, and prosthetic foot (*p* > 0.05). Statistically significant differences were observed between the groups with respect to stump length, percentage of the residual limb, number of prostheses used to date, duration of use of the current prosthesis (years), and suspension systems (*p* < 0.05).

### 3.3. Comparisons Among Three Groups

**Figure 3:**
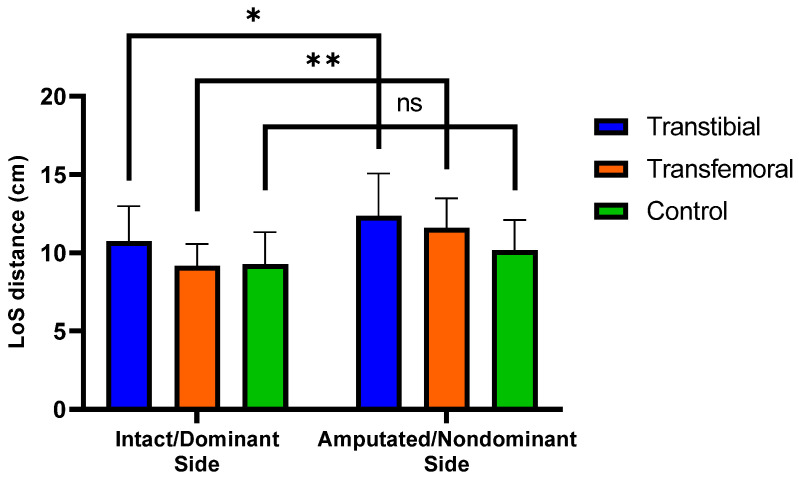
Significant differences (0.01 < *p* < 0.05) are indicated by an asterisk (*). Significant differences (*p* < 0.01) are indicated by an asterisk (**). “ns” represents non-significant *p*-values. LoS represents Limits of Stability. Mean (±SD) LoS distances intact/dominant and amputated/nondominant sides across the three groups.

In the anteroposterior and lateral postural sway ranges CSEC and lateral postural sway range NSEC, the highest values were observed in the TFA group, whereas the lowest values were found in the CG, with a statistically significant difference among the three groups (*p* < 0.05). The differences observed in the anteroposterior postural sway range CSEC (mean difference = 0.35 cm, 95% CI: 0.02 to 0.69, Tukey *p* = 0.037) and in the lateral postural sway range NSEC (mean difference = 0.14 cm, 95% CI: 0.04 to 0.24, Tukey *p* = 0.005) were due to the pairwise comparisons between TFA group and CG. No significant differences were observed among the three groups with respect to other postural control values (*p* > 0.05) (Table 3 and Supplementary Table S1). Significant differences were observed in the within-group comparisons of LoS distance values between IS and AS for both the TTA and TFA groups (*p* < 0.05) (Figure 3). It was determined that the muscle activations assessed during forward and backward perturbations were similar on the IS of the TTA and TFA groups and on the DS of the CG (*p* > 0.05) (Table 3).

### 3.4. Comparisons Between Transtibial and Control Group

The two-way mixed-design ANOVA revealed no significant group × side interaction effect for any of the muscle activation variables (*p* > 0.05). In the forward perturbation condition, significant main effects of group were found for the activation of the RF and BF muscles, and a significant main effect of side was observed for BF activation (*p* < 0.05). However, the main effect of side for RF activation during forward perturbation was not significant (*p* > 0.05). In the backward perturbation condition, a significant main effect of side was observed for RF activation (*p* < 0.05), whereas no significant main effects of group or side were found for the other muscle activations (*p* > 0.05).

In the TTA group, a significant difference was observed between the IS and AS in biceps femoris muscle activation during forward perturbation (mean difference = −24.17, 95% CI: −45.72 to −2.63, Bonferroni *p* = 0.030). Additionally, there was a significant difference between the TTA group and CG in RF muscle activation on the AS/NDS during forward perturbation (mean difference = 34.73, 95% CI: 0.44 to 69.01, Bonferroni *p* = 0.047) (Table 4 and Supplementary Table S2).

**Figure 4:**
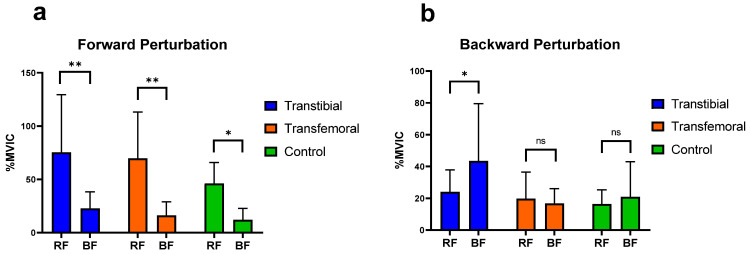
Significant differences (0.01 < *p* < 0.05) are indicated by an asterisk (*). Significant differences (*p* < 0.01) are indicated by an asterisk (**). “ns” represents non-significant *p*-values. RF: Rectus Femoris, BF: Biceps Femoris. %MVIC: Percentage of maximum voluntary isometric contraction. (**a**) Mean (±SD) %MVIC values of the thigh muscles on the intact/dominant side during forward perturbations. (**b**) Mean (±SD) %MVIC values of the thigh (knee) muscles on the intact/dominant side during backward perturbations.

**Figure 5:**
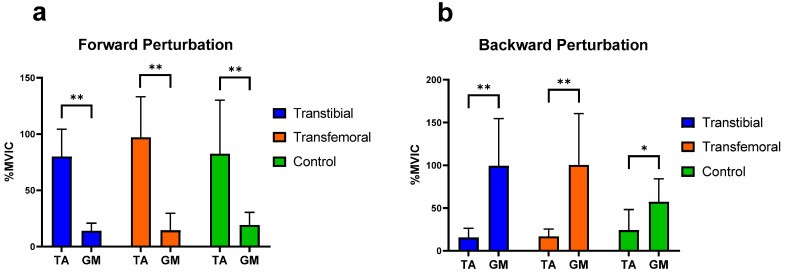
Significant differences (0.01 < *p* < 0.05) are indicated by an asterisk (*). Significant differences (*p* < 0.01) are indicated by an asterisk (**). TA: Tibialis anterior, GM: Medial head of gastrocnemius. %MVIC: Percentage of maximum voluntary isometric contraction. (**a**) Mean (±SD) %MVIC values of the leg muscles on the intact/dominant side during forward perturbations. (**b**) Mean (±SD) %MVIC values of the leg muscles on the intact/dominant side during backward perturbations.

It was concluded that, during forward perturbations, the differences in the activation of RF and BF muscles of the thigh, as well as the TA and GM muscles of the calf, were statistically significant within each group (*p* < 0.05). During backward perturbations, the differences in the activation of TA and GM muscles were found to be significant within each group (*p* < 0.05), whereas the differences in the activation of the RF and BF muscles were significant only in the TTA group (*p* < 0.05) (Figure 4 and Figure 5).

## 4. Discussion

In our study investigating static, predictive, and reactive postural responses in individuals with TTA, TFA, and healthy controls, the most notable finding regarding static postural reactions was that, under conditions with restricted visual input, significant differences in postural sway were primarily observed between TFAs and controls. In terms of predictive postural responses, the LoS values were similar across all directions among the groups; however, in individuals with amputation, LoS assessments in the lateral directions revealed that displacement toward the AS was greater than that toward the IS. Regarding reactive postural responses, muscle activation patterns of agonist and antagonist muscles changed in relation to the direction of surface perturbations (except for backward perturbations in TFAs), and the TTAs exhibited greater activation of thigh muscles than the CG during forward perturbations.

It was important that the study groups were similar in terms of demographic characteristics so that the findings could be attributed to differences related to the amputation level. The difference in the percentage of residual limbs between TTAs and TFAs, considering amputation and prosthesis characteristics, is a natural consequence of the difference in amputation level. Nevertheless, reporting the stump length for each group was considered to contribute to the interpretation of the results, although a direct comparison of stump lengths between different amputation levels was not intended. In this context, a longer stump length in TFAs does not provide an advantage over TTAs, nor does the relatively shorter stump length in TTAs obscure the functional advantage of knee joint preservation in TTAs. Furthermore, the similarity of the groups in terms of amputation age, duration of prosthesis use, and duration of daily prosthesis use is essential to eliminate potential differences in prosthetic adaptation and function, allowing the results to be interpreted independently of these confounding factors.

Molero-Sánchez et al. reported that in individuals with traumatic unilateral TTA, the maximum CoG excursion among the LoS parameters was lower in the anterior, posterior, IS, and AS directions than in healthy controls; however, this difference reached only marginal statistical significance in the posterior direction, with no significant differences observed in the other directions [24]. Although no significant differences were observed between the groups in terms of LoS parameters in our study, only the anterior LoS distance was lower in the TTA and TFA groups than in the control group. In our study, positioning the feet symmetrically and in a preferred comfortable stance during the LoS test may have led to an expansion of the base of support (BoS) and an increase in values in amputees. The findings of Fernie et al., who reported significantly greater BoS in TTAs and TFAs than in controls [25], support our interpretation that amputee individuals may exhibit a wider BoS than the CG. Moreover, our results demonstrated significantly greater LoS distances in the AS direction than in the IS direction in both TTAs and TFAs. One possible explanation for this outcome is that amputees typically exhibit an asymmetrical stance by shifting their body weight toward the intact limb [26,27,28,29,30], thereby causing the CoP to shift toward the IS. Under these conditions, the position of the CoP restricts the movement of the LoS toward the intact side (IS), while increasing the distance it can move toward the AS. Another contributing factor may be that, as the intact limb is primarily used for stability, support is obtained from the intact limb during movements toward the AS; however, sufficient support cannot be provided by the prosthetic limb during movements toward the IS [31].

Buckley et al. demonstrated that individuals with lower-limb amputation exhibited significantly greater CoP excursion values than able-bodied individuals in both the anteroposterior and mediolateral directions during standing on a stable surface with eyes open [32]. Conversely, in our study, no significant differences were observed among the three groups regarding the anteroposterior and lateral postural sway ranges under NSEO conditions. The differing results may be attributed to the type of knee joint used by individuals with TFAs. Buckley et al. evaluated amputee individuals using hydraulic knee joints, whereas our study included those using microprocessor-controlled knee joints. The findings of Kaufman et al. [33], demonstrating that microprocessor-controlled knee joints improve balance in TFAs compared with hydraulic joints, also support our inference. For the TTAs, differences in socket design may explain the inconsistent findings. Buckley et al. used patellar tendon bearing full contact sockets, whereas the TTAs in our study utilized total surface bearing sockets. The results of Yiğiter et al., who reported that weight transfer on the AS was greater and more comparable to normal in total surface-bearing sockets than in patellar tendon-bearing designs [34], support our interpretation that the differing outcomes in TTAs are socket-related.

In our study, no significant differences were observed among the three groups in the anteroposterior and lateral postural sway ranges under CSEO conditions, whereas significant differences were observed when visual information was restricted (except for the anteroposterior postural sway range under NSEC), highlighting the important role of visual input in postural control. Post hoc pairwise comparisons (anteroposterior postural sway range–CSEC, lateral postural sway range–NSEC) revealed greater reliance on visual than on somatosensory input in TFAs compared with the CG. The greater dependence on visual input may be explained by the reduced somatosensory feedback from the AS in TFAs, which exerts a more disruptive effect on postural control when visual cues are limited [35]. Additionally, Claret et al. reported that restricting visual input in TFAs increases reliance on proprioceptive mechanisms, particularly in controlling the intact limb [36].

Our study demonstrated that there were no significant differences in IS/DS muscle activation among the groups during forward and backward surface perturbations.

In their study evaluating the automatic postural reactions of healthy individuals on a normal support surface, Horak and Nashner concluded that the TA and RF muscles were more active during forward surface perturbations, while the GM and BF muscles were more active during backward perturbations [37]. Consistent with this, our study similarly demonstrated that during forward perturbations, the RF in the thigh region and the TA in the lower leg region exhibited significantly greater activation than the BF and GM, respectively, across all groups. This finding suggests that, with respect to the muscles we evaluated, the muscular responses of individuals with amputation to forward perturbations are comparable to the postural responses observed in healthy control individuals. During backward perturbations, in line with the findings of Horak and Nashner, our study also showed that the GM was significantly more activated than the TA in all groups; however, only in the TTA group was the BF significantly more activated than the RF. Furthermore, among the three groups, TFAs exhibited the lowest BF activation during backward perturbations. This finding suggests that TFAs may employ different postural mechanisms than TTAs during such perturbations.

The results of this study showed that during both forward and backward perturbations, thigh muscle amplitudes on the IS and AS in individuals with TTA were higher than those on the DS and NDS of the controls, respectively, and that the main effect of group was significant for the RF and BF muscles during forward perturbations. Rusaw et al. also reported that TTAs show different postural adaptation and compensation mechanisms compared with the CG when standing on a sway-referenced support surface [38], supporting our results. In TTAs, greater support from the thigh muscles may be required to compensate for factors such as sensory impairment on the AS, loss of leg muscles, and an upward shift of the CoM compared with healthy individuals. In addition, our study showed that in individuals with TTA, RF muscle activation on the AS was significantly higher than that on the NDS of the CG. It has been reported that in individuals with TTA, sensory impairments in the AS, reduced knee extension moments, and asymmetrical load distribution [39,40], together with accompanying neuromuscular adaptations and reduced cortical inhibition [41,42], lead to a decrease in the cross-sectional area of the RF muscle and consequent quadriceps weakness [40,43]. This process may result in reflex inhibition and functional weakening of the extensors of the AS. Therefore, these structural and neurophysiological alterations affecting the RF muscle in individuals with TTA may have necessitated the maintenance of postural control through higher-amplitude muscle activations during forward perturbations. The findings obtained from this study provide important insights into the postural control responses of TTAs and TFAs. These insights are expected to make valuable contributions to rehabilitation practices. However, our study had certain limitations.

Firstly, the sample size was small and slightly imbalanced. This is an inherent methodological limitation, understandable given the substantial difficulty and stringent criteria required to obtain a homogeneous cohort of high-functioning amputees (K3/K4). Consequently, the restricted sample size and specialized nature of the participants limit the generalizability of our findings to the broader amputee population. The findings of this study are limited to young and adult individuals with traumatic amputations; therefore, the results cannot be generalized to individuals with amputations of other etiologies. Although the participants were instructed to position their feet equidistant from the midline and as symmetrically as possible, the fact that they were allowed to choose a comfortable stance may have influenced the standardization of the postural control measurements. Furthermore, the absence of whole-body kinetic and kinematic assessments, including the trunk and upper extremities, restricted the interpretation of findings related to these segments to a hypothetical level. These factors should be considered among the limitations of the present study.

## 5. Conclusions

To the best of our knowledge, this study represents the first to investigate the effects of unexpected surface perturbations on lower extremity muscle activation in individuals with transtibial and transfemoral amputations. The findings suggest that postural control responses in individuals with lower limb amputation may differ according to the level of amputation. In individuals with transfemoral amputations, increased dependence on visual input during quiet standing was observed, whereas individuals with transtibial amputations appeared to rely more on thigh muscles during surface perturbations. In conclusion, to improve postural control in individuals with transtibial and transfemoral amputations, rehabilitation programs should place greater emphasis on approaches that incorporate exercises targeting both the amputated and intact sides, specifically addressing static, predictive, and reactive balance components, together with sensory integration training.

## Figures and Tables

**Table 1:** General physical characteristics of transtibial, transfemoral, and control groups.

Characteristics		Transtibial *(n* = 10)	Transfemoral (*n* = 9)	Control (*n* = 10)	
		*n* (%)	*n* (%)	*n* (%)	*p*_α_
Sex	Male	7 (70.0)	9 (100.0)	8 (80.0)	0.322
	Female	3 (30.0)	-	2 (20.0)	
		**X ± SD**	**X ± SD**	**X ± SD**	***p*_β_**
Age [years]		32.50 ± 9.26	33.11 ± 6.41	32.00 ± 7.60	0.954
Body height [m]		1.74 ± 0.08	1.74 ± 0.05	1.74 ± 0.06	0.988
Body mass [kg]		78.20 ± 11.44	74.70 ± 13.18	72.09 ± 11.80	0.536
Body mass index [kg/m^2^]		25.87 ± 3.95	24.70 ± 4.01	23.79 ± 3.22	0.469
Intact/Dominant limb length [cm]		89.55 ± 6.08	88.67 ± 4.36	88.90 ± 4.12	0.921
Residual/Nondominant limb length [cm]		66.00 ± 6.35	35.44 ± 8.53	89.00 ± 4.06	<0.001 *

*n*: Number of participants, X: Mean, SD: Standard deviation. *p*_α_: Monte Carlo exact test is the *p* value. *p*_β_: One-way ANOVA test is the *p* value. Significant differences between groups are indicated by bolded *p* values. *: The observed difference was due to the pairwise comparisons between transtibial and transfemoral, transtibial and control, transfemoral and control.

**Table 2:** Amputation and prosthesis related data.

		Transtibial (*n* = 10)	Transfemoral (*n* = 9)	
		X ± SD	X ± SD	*p*_δ_
Stump length [cm]		17.00 ± 5.16	35.44 ± 8.53	**0.001**
Percentage of residual limb (%)		73.66 ± 4.28	39.97 ± 9.60	**<0.001**
Amputation age [years]		18.70 ± 8.11	22.56 ± 6.27	0.388
Duration of prosthesis use [years]		13.50 ± 7.81	9.78 ± 4.15	0.220
Number of prostheses used to date		5.10 ± 3.45	2.33 ± 1.41	**0.032**
Duration of use of the current prosthesis [years]		1.95 ± 1.42	4.61 ± 1.76	**0.003**
Duration of daily prosthesis use [hours]		13.95 ± 2.23	13.33 ± 2.51	0.769
		***n* (%)**	***n* (%)**	***p*_η_**
Amputation side	Right	8 (80.0)	5 (55.6)	0.350
	Left	2 (20.0)	4 (44.4)	
Suspension systems	Pin system	1 (10.0)	3 (33.3)	0.003 *
	Passive vacuum	2 (20.0)	6 (66.7)	
	Active vacuum	7 (70.0)	-	
Prosthetic foot	Double axis foot	1 (10.0)	-	1.000
	Carbon foot	8 (80.0)	9 (100.0)	
	Hydraulic carbon foot	1 (10.0)	-	
Types of Microprocessor Knee	Rheo Knee	-	2 (22.2)	-
	Genium	-	4 (44.4)	
	C-leg	-	3 (33.3)	

*n*: Number of participants, X: Mean, SD: Standard deviation. *p*_δ_: Mann–Whitney U test is the *p* value. *p*_η_: Fisher’s or Monte Carlo exact test, as appropriate, is the *p* value. Significant differences between groups are indicated by bolded *p* values. *: The observed difference was due to the active vacuum system.

**Table 3:** Postural control and muscle activation (intact/dominant side) measurements.

		Transtibial (*n* = 10)	Transfemoral (*n* = 9)	Control (*n* = 10)	*p*_β_	η^2^
Postural Control		X ± SD	X ± SD	X ± SD		
LoS distance (cm)	Anterior	7.72 ± 1.04	7.43 ± 1.37	8.72 ± 2.03	0.173	0.126
	Posterior	5.82 ± 1.54	5.14 ± 1.52	5.42 ± 1.68	0.645	0.033
	IS/DS	10.75 ± 2.24	9.17 ± 1.41	9.27 ± 2.06	0.154	0.134
	AS/NDS	12.37 ± 2.71	11.61 ± 1.88	10.17 ± 1.93	0.100	0.163
Anteroposterior postural sway range (cm)	NSEO	0.50 ± 0.14	0.44 ± 0.15	0.50 ± 0.17	0.629	0.034
	NSEC	0.73 ± 0.21	0.90 ± 0.25	0.68 ± 0.16	0.068	0.187
	CSEO	0.71 ± 0.27	0.55 ± 0.15	0.65 ± 0.29	0.378	0.072
	CSEC	1.15 ± 0.29	1.31 ± 0.37	0.96 ± 0.21	0.047 *	0.210
Lateral postural sway range (cm)	NSEO	0.25 ± 0.08	0.26 ± 0.09	0.22 ± 0.10	0.594	0.041
	NSEC	0.32 ± 0.07	0.39 ± 0.11	0.25 ± 0.08	0.007 *	0.321
	CSEO	0.60 ± 0.21	0.46 ± 0.24	0.48 ± 0.27	0.415	0.065
	CSEC	0.70 ± 0.14	0.74 ± 0.28	0.51 ± 0.18	**0.049**	0.207
**Muscle activation (IS/DS)**		**M IQR (25–75)**	**M IQR (25–75)**	**M IQR (25–75)**	***p*_¶_**	**η^2^(H)**
Forward perturbation	RF	55.69 (46.04–99.33)	48.68 (39.37–110.03)	39.87 (32.24–58.67)	0.173	0.058
	BF	19.02 (9.14–39.95)	8.36 (7.16–31.14)	8.03 (5.42–15.63)	0.113	0.091
	TA	83.03 (60.57–99.40)	77.55 (68.77–119.91)	69.27 (57.59–100.02)	0.459	<0.01
	GM	11.98 (8.05–17.49)	10.57 (5.52–15.29)	15.79 (11.25–21.96)	0.135	0.077
Backward perturbation	RF	22.28 (13.64–36.27)	19.10 (7.67–24.32)	14.99 (9.35–24.87)	0.404	<0.01
	BF	38.08 (13.08–81.83)	13.46 (9.58–24.91)	15.18 (5.56–24.50)	0.201	0.047
	TA	12.90 (6.25–26.18)	15.28 (11.47–23.56)	14.03 (4.68–38.73)	0.843	<0.01
	GM	77.64 (49.74–152.42)	83.97 (67.95–167.76)	51.17 (33.12–75.94)	0.105	0.096

*n*: Number of participants, X: Mean, SD: Standard deviation, M: Median, IQR: Interquartile range. LoS: Limits of stability, IS: Intact side, DS: Dominant side, AS: Amputated side, NDS: Nondominant side, NSEO: Normal surface eyes open, NSEC: Normal surface eyes closed, CSEO: Compliant surface eyes open, CSEC: Compliant surface eyes closed. RF: Rectus femoris, BF: Biceps femoris, TA: Tibialis anterior, GM: Medial head of gastrocnemius. *p*_β_: One-way ANOVA is the *p* value, *p*_¶_: Kruskal–Wallis test is the *p* value. Significant differences between groups are indicated by bolded *p* values. *: The observed differences were due to the pairwise comparison between transfemoral and control. η^2^: Effect size (eta-squared) for one-way ANOVA; η^2^(H): Effect size (eta-squared based on the Kruskal–Wallis H statistic). Negative η^2^(H) values resulting from the Kruskal–Wallis test were considered negligible and are reported as <0.01.

**Table 4:** Muscle activation measurements of transtibial and control groups.

		Transtibial (*n* = 10)			Control (*n* = 10)			Transtibial vs. Control		Group Effect	Side Effect	Interactions
Perturbation Direction	Muscle Activation	IS	AS	*p*	DS	NDS	*p*	IS vs. DS	AS vs. NDS			
		X ± SD	X ± SD		X ± SD	X ± SD		*p*	*p*			
Forward	RF	75.43 ± 54.09	82.46 ± 36.85	0.636	46.10 ± 19.72	47.74 ± 36.12	0.912	0.125	**0.047**	**0.033**	0.680	0.797
	BF	22.71 ± 15.66	46.88 ± 39.27	**0.030**	12.13 ± 10.72	20.97 ± 21.96	0.400	0.095	0.085	**0.038**	**0.035**	0.304
Backward	RF	24.04 ± 13.84	41.94 ± 42.82	0.114	16.38 ± 8.96	32.45 ± 24.34	0.154	0.159	0.550	0.341	**0.039**	0.906
	BF	43.46 ± 36.09	33.77 ± 20.87	0.115	20.95 ± 22.10	20.84 ± 16.90	0.985	0.110	0.145	0.106	0.251	0.262

*n*: Number of participants, IS: Intact side, DS: Dominant side, AS: Amputated side, NDS: Nondominant side, vs: Versus, X: Mean, SD: Standard deviation, RF: Rectus femoris, BF: Biceps femoris. A two-way mixed-design ANOVA is the *p* value. Significant differences between groups are indicated by bolded *p* values.

## Data Availability

The data presented in this study are available on request from the corresponding author.

## Supplementary Materials

The following supporting information can be downloaded at: https://www.mdpi.com/article/10.3390/jcm14248737/s1, Table S1. Pairwise group comparisons with mean differences and 95% confidence intervals for postural control parameters showing significant group effects; Table S2. Pairwise comparisons with mean differences and 95% confidence intervals for muscle activation (based on estimated marginal means).

## Abbreviations

The following abbreviations are used in this manuscript:

TTA	Transtibial amputation
TFA	Transfemoral amputation
CG	Control group
RF	Rectus femoris
BF	Biceps femoris
TA	Tibialis anterior
GM	Medial head of the gastrocnemius
CoM	Center of mass
BoS	Base of support
CoP	Center of pressure
LoS	Limits of Stability
NSEO	Normal surface eyes open
NSEC	Normal surface eyes closed
CSEO	Compliant surface eyes open
CSEC	Compliant surface eyes closed
IS	Intact side
AS	Amputated side
DS	Dominant side
NDS	Nondominant side
MVIC	Maximum voluntary isometric contraction
RMS	Root Mean Square
vs	Versus
