# Supplementary material for: Comparative Analysis of Standing Postural Control and Perturbation-Induced Muscle Activity in Transtibial and Transfemoral Amputees
Source: J Clin Med. 2025 Dec 10;14(24):8737. doi: 10.3390/jcm14248737 (PMC12734143; doi:10.3390/jcm14248737)
Supplement: Supplementary file 1 [file jcm-14-08737-s001.zip › jcm-4004456-supplementary.pdf]

Supplementary Table S1. Pairwise group comparisons with mean differences and 95% confidence intervals for postural control parameters showing significant group effects

Postural Control	Pairwise Comparison	Mean Difference	95% CI Lower	95% CI Upper	Tukey p-value
Anteroposterior postural sway range (cm)- CSEC	Transfemoral vs Control	0.35	0.02	0.69	**0.037**
Lateral postural sway range (cm)- NSEC	Transfemoral vs Control	0.14	0.04	0.24	**0.005**

CSEC: Compliant surface eyes closed, NSEC: Normal surface eyes closed. Mean differences and 95% confidence intervals (CI) were obtained from Tukey HSD post-hoc tests following significant one-way ANOVA results. Positive mean differences indicate higher values in the transfemoral group compared with control group. Only statistically significant pairwise comparisons (Tukey $p<0.05$) are reported.

Supplementary Table S2. Pairwise comparisons with mean differences and 95% confidence intervals for muscle activation (based on estimated marginal means)

Perturbation Direction	Muscle Activation	Pairwise Comparison	Mean Difference	95% CI Lower	95% CI Upper	Bonferroni p-value
Forward	RF	Transtibial (AS) vs Control (NDS)	34.73	0.44	69.01	**0.047**
Forward	BF	IS vs AS (Transtibial)	-24.17	-45.72	-2.63	**0.030**

Mean differences and 95% confidence intervals (CI) were obtained from Bonferroni-adjusted pairwise comparisons based on estimated marginal means from the two-way mixed-design ANOVA. Only statistically significant pairwise comparisons are reported. IS: intact side, AS: amputated side, NDS: nondominant side, RF: rectus femoris, BF: biceps femoris.
